# Sex‐specific recruitment rates contribute to male‐biased sex ratio in Adélie penguins

**DOI:** 10.1002/ece3.10859

**Published:** 2024-02-20

**Authors:** Virginia Morandini, Katie M. Dugger, Annie E. Schmidt, Arvind Varsani, Amélie Lescroël, Grant Ballard, Phil O'B. Lyver, Kerry Barton, David G. Ainley

**Affiliations:** ^1^ Oregon Cooperative Fish and Wildlife Research Unit, Department of Fisheries and Wildlife Oregon State University Corvallis Oregon USA; ^2^ Migres Foundation CIMA Tarifa Spain; ^3^ U.S. Geological Survey, Oregon Cooperative Fish and Wildlife Research Unit, Department of Fisheries and Wildlife Oregon State University Corvallis Oregon USA; ^4^ Point Blue Conservation Science Petaluma California USA; ^5^ The Biodesign Center for Fundamental and Applied Microbiomics, Center for Evolution and Medicine, School of Life Sciences Arizona State University Tempe Arizona USA; ^6^ Manaaki Whenua Landcare Research New Zealand Ltd. Lincoln New Zealand; ^7^ H.T. Harvey & Associates Ecological Consultants Los Gatos California USA

**Keywords:** Adélie penguin, adult sex ratio (ASR), demographics, recruitment, seabird, sex‐specific, survival

## Abstract

Sex‐related differences in vital rates that drive population change reflect the basic life history of a species. However, for visually monomorphic bird species, determining the effect of sex on demographics can be a challenge. In this study, we investigated the effect of sex on apparent survival, recruitment, and breeding propensity in the Adélie penguin (*Pygoscelis adeliae*), a monochromatic, slightly size dimorphic species with known age, known sex, and known breeding history data collected during 1996–2019 (*n* = 2127 birds) from three breeding colonies on Ross Island, Antarctica. Using a multistate capture–mark–recapture maximum‐likelihood model, we estimated apparent survival ($\hat{S}$), recapture (resighting) probability ($\hat{p}$), and the probability of transitioning among breeding states and moving between colonies ($\hat{\psi }$; colony‐specific non‐juvenile pre‐breeders, breeders, and non‐breeders). Survival rate varied by breeding status and colony, but not sex, and pre‐breeders had higher survival rates than breeders and non‐breeders. Females had a higher probability of recruiting into the breeding population each year and may enter the breeding pool at younger ages. In contrast, both sexes had the same probability of breeding from year to year once they had recruited. Although we detected no direct sex effects on survival, the variation in recruitment probability and age‐at‐first reproduction, along with lower survival rates of breeders compared to pre‐breeders, likely leads to shorter lifespans for females. This is supported by our findings of a male‐biased mean adult sex ratio (ASR) of 1.4 males for every female ($\hat{x}$ proportion of males = 0.57, SD = 0.07) across all colonies and years in this metapopulation. Our study illustrates how important it can be to disentangle sex‐related variation in population vital rates, particularly for species with complex life histories and demographic dynamics.

## Graphical abstract

We detected no survival differences between male and female Adelie penguins, but the probability of recruiting into the breeding population was higher for females. In conjunction with the observed decrease in survival for birds of both sexes once they began to breed, females likely have shorter lifespans than males. These vital rate differences likely contributed to the male‐biased sex ratio we observed in our study.

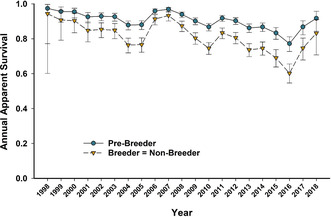

## 1 INTRODUCTION

Variation in vital rates that drive population change have been studied in a wide range of bird and mammal species (e.g., Dahlgren et al., 2016; Dugger et al., 2016; Forrester & Wittmer, 2013; Hostetler et al., 2021), revealing sex‐related differences in basic biology and ecology (Clutton‐Brock et al., 2002; Eberhart‐Phillips et al., 2018). Sex‐related differences in annual survival have been well‐documented among avian taxa exhibiting a range of life‐history strategies (e.g., Ferrer & Hiraldo, 1992; Grüebler et al., 2008; Oro et al., 2018). Often the sex investing the most time and energy in offspring is exposed to a higher risk of mortality resulting in lower annual survival (Lack, 1968; Payevsky, 2021). In species where sexes differ in size, lower survival is often reported in the smaller sex (Martínez‐Abraín et al., 2006; Vanstreels et al., 2013), although the opposite pattern (Deakin et al., 2019), or no relationship (Oro et al., 2010) has also been reported. For many monomorphic or slightly size dimorphic bird species (like many seabirds), the potential effect of sex on demographics is often ignored as it is difficult to investigate (but see Ainley & DeMaster, 1980; Gownaris & Boersma, 2019). However, sex‐related variation in survival as nestlings, juveniles, or adults can result in a skewed adult sex ratios (ASR; measured relative to the proportion of breeding‐aged males; Székely et al., 2014), a fundamental variable in demography and population biology (Donald, 2007; Székely, Liker, et al., 2014). Across the spectrum of avian taxa, sex ratio patterns are strongly associated with social mating systems (e.g., monogamy, polygamy, polyandry, etc.) and sex‐specific mating behavior within those systems including mate choice, parental care, and duration of pair‐bonds (e.g., Liker et al., 2014; Székely, Liker, et al., 2014).

Stochastic variation of sex ratios in wild bird populations on decadal time scales has been less well‐studied, but can cause mating system shifts (Kus et al., 2017), influence mate selection behavior (Madden & Whiteside, 2013), and the prevalence of extra‐pair mating in monogamous systems (Arrieta et al., 2022; Grant & Grant, 2019). Sex ratio variation can also increase mate‐competition (e.g., Ewen et al., 2011), and decrease the duration of long‐ and short‐term pair bonds (Eberhart‐Phillips et al., 2018; Maness & Anderson, 2007; Pilastro et al., 2001). Thus, sex‐based variation in population vital rates (e.g., survival, productivity, dispersal, recruitment) that generates changes in ASR and/or competition for mates among breeders, can ultimately affect demography at the population level (e.g., Gownaris & Boersma, 2019; Morandini et al., 2019), particularly for small populations (Bessa‐Gomes et al., 2004).

The Adélie penguin (*Pygoscelis adeliae*) is a monochromatic species that exhibits mild sexual size dimorphism, with adult males averaging slightly larger than females in mass (mean ± SD = 4.5 ± 0.6 kg vs. 4.2 ± 0.5 kg), flipper length (187.3 ± 6.1 mm vs. 180.6 ± 6.2 mm), and bill length (24.2 ± 1.5 mm vs. 22.7 ± 1.5 mm; AS, GB unpublished data). The species has relatively low annual survival compared to other seabirds (e.g., Ainley & DeMaster, 1980; Ballerini et al., 2009; Emmerson & Southwell, 2011), exhibits highly variable delayed maturation (Kappes et al., 2021) and without large‐scale environmental disturbance, high breeding site fidelity (Dugger et al., 2010). As one of the most well‐studied avian species in the world (Ainley, 2002) and a designated “indicator species” by agencies charged with managing resource extraction in the Southern Ocean (Agnew, 1997), many aspects of Adélie penguin natural history are well‐understood. However, as a monochromatic species that can be difficult to sex in the field, very few studies of Adélie penguins have investigated sex‐related demographic differences. When it has been evaluated, the focus has been on annual survival, and males had slightly higher survival rates than females (Ainley & DeMaster, 1980; Dugger et al., 2006). Sex‐related differences in other potentially important vital rates, including the probability of recruiting into the breeding population and the probability that a breeding bird takes a sabbatical in the subsequent year (i.e., breeding propensity) have never been reported in the literature for this species.

We initiated this study to evaluate the validity of previous findings regarding sex‐specific differences in apparent survival (Ainley & DeMaster, 1980, Dugger et al., 2006), while also accounting for potential survival differences relative to reproductive state. In addition, we wanted to understand how sex influences other important vital rates including the probability of recruitment and breeding propensity. To address these questions, we collected longitudinal data on a sample of known‐age, known‐sex, known‐breeding history Adélie penguins during 1996–2019 (*n* = 2127 birds) within a cluster of three breeding colonies of vastly different size on Ross Island, Antarctica. These three colonies along with a fourth in the vicinity, comprised a functioning metapopulation (~11% of global population; Lynch & LaRue, 2014) where we have quantified detectable immigration/emigration rates (Dugger et al., 2010, 2014; LaRue et al., 2013). Field observations at these colonies suggested that the ASR was skewed toward males, consistent with earlier findings for the largest colony (Ainley & DeMaster, 1980), but the potential bias in the assignment of sex from field observations has not been evaluated. Male behaviors generally make them more “detectable” (i.e., more likely to be noticed and sexed by field observers than females). For this reason, estimating sex‐related differences in demographic rates requires accounting for detection rates <1.0 and correcting for differences in detection rates between the sexes. We attempted to address these issues by evaluating our assignment of sex based on field observations for non‐juvenile banded birds through comparison of those field‐based assignments to the “true” sex of a subset of birds we sexed genetically. We used a multistate capture–mark–recapture model framework to test for sex‐related differences in apparent survival ($\hat{S}$), and transition rates ($\hat{\Psi }$) that reflected the probability of recruiting into the breeding population, breeding propensity after recruitment, and movement rates between colonies, while accounting for sex‐related variation in resighting probability ($\hat{p}$).

## 2 MATERIALS AND METHODS

Adélie penguin chicks were marked with a hydrodynamically shaped, individually numbered stainless‐steel band (see Dugger et al., 2006 for details on band design) on the left flipper at three adjacent colonies (following the definition in Santora et al., 2020) on Ross Island, Antarctica: Cape Royds (77°33′ S, 166°10′ E), Cape Crozier (77°27′ S, 169°14′ E), and Cape Bird (77°13′ S, 166°28′ E). Bands were affixed to chicks just prior to fledging each austral summer from 1994–1995 to 2019–2020. Colony size ranged by orders of magnitude from Cape Royds, the smallest colony (~2–4 K breeding pairs), to the medium‐sized Cape Bird (~40–75 K), and the largest colony, Cape Crozier (~150–300 K; Lyver et al., 2014). We searched each Ross Island colony for banded birds every 2–7 days, November through January each year. When a banded bird was observed, we read the band number through binoculars and recorded breeding status. When ≥1 egg or chick was observed, we marked the nest with GPS coordinates using a nail and a cattle‐tag placed ~20 cm from the nest edge. We then followed marked nests throughout the breeding season to determine reproductive outcomes. Sex was assigned opportunistically during band resighting using a combination of observed behaviors (ecstatic displays for males (Marks et al., 2010), copulation position, muddy tread marks on an individual's back, incubation timing early in the season) and comparisons of size when both members of the pair were present (see Ainley, 2002). When a bird's sex was recorded by multiple observers across multiple years, we used all records to make the final sex assignment. If a bird was ever observed copulating, we used that observation to assign sex. Otherwise, we totaled the number of times (within and across all years) that a bird was recorded as a male and all the times it was recorded as a female to assign sex. For example, if a bird was assigned as “male” at least twice and ≥3 times more than it was recorded as “female,” it was assigned as “male.” If the sex could not be assigned given these rules, we excluded the bird from this analysis. To evaluate the success (% assigned correctly) of our sex assignments, we used a two‐sided exact binomial test (Fay, 2010) to compare the sex assignment of a subset of birds from field observations during 2010–2013 (*n* = 140 individuals) to their sex based on DNA extracted from feathers for these same birds. We used DNA extracted from the feathers as a template for a CHD‐gene targeted PCR with primer pair 2550F (5′‐GTT ACT GAT TCG TCT ACG AGA‐3′) and 2718R (5′‐ATT GAA ATG ATC CAG TGC TTG‐3′) (Fridolfsson & Ellegren, 1999).

### 2.1 Demographic analyses

We created encounter histories for each bird of known sex that represented multiple resightings collapsed within a year into a single resighting history that characterized reproductive state (i.e., pre‐breeder, breeder, non‐breeder) and colony where the bird was observed. All birds included in this study were banded as chicks, but they cannot be sexed at that age, and we could not assume 1:1 sex ratio in our annual banded sample of fledglings. In addition, Adélie penguins exhibit highly variable delayed maturation and don't return to a breeding colony until at least 2 years of age (i.e., yearlings were rarely seen during this study and banded yearlings were never observed), with many birds not returning until they are 4–5 years old (Ainley et al., 1983). Thus, during their first few years prior to a colony visit, birds are “unobservable,” and we could only determine sex for birds that eventually returned to the colony as pre‐breeders or breeders (≥2 years old). For this reason, only encounter histories for birds that returned to either of the three colonies after the year they fledged were included in the analysis, and that first resighting after banding was the first time a bird was “released” and available for subsequent resighting. We also excluded the very earliest years when our banded population was small and all birds were unobservable juveniles or pre‐breeders, so the time series used in this analysis was 1998–2019 (22 years). We encountered a total of 2127 post‐juvenile birds (≥2 years of age; 1236 males and 891 females) that eventually were assigned a sex at some point during this time period using our visual sex‐assignment criteria.

We used a multistate capture–mark–recapture model (White et al., 2006) to generate maximum‐likelihood estimates of apparent survival (S), probability of resighting (p), and the probability of transitioning between states (Ψ) that included combinations of year‐specific reproductive states and colony locations. We coded sex as a binary individual covariate for each bird with males coded as “1” and females as “0”. We classified reproductive status into three states: (1) “pre‐breeders” (PR) were birds that did not have an egg or chick in the current year and had not been recorded with an egg or chick in any previous year, (2) “breeders” (BR) were birds observed with ≥1 egg or chick in the current year, and (3) “non‐breeders” (NB) were birds not observed with an egg or chick in the current year but classified as a breeder one or more previous years. This resulted in a combination of three reproductive states at each of the three colony locations, for a total of nine different states (e.g., Royds pre‐breeder, Royds breeder, Royds non‐breeder, Bird pre‐breeder, Bird breeder, etc.). Between years, individuals could move between pre‐breeder states or transition from pre‐breeder to breeder (i.e., recruit into the breeding population) while either staying at the colony of origin or moving to a different colony. In addition, breeders and non‐breeders could transition back and forth between these two states and move among colonies. However, pre‐breeders could not become non‐breeders, and breeders and non‐breeders could not go back to the pre‐breeder state. Thus, although we had 72 different possible transitions between these nine states, 9 were derived through subtraction and 27 (more than a third) were not biologically possible so we fixed them to zero (Table S1). We observed high site fidelity for breeding birds between subsequent seasons, with much lower transition probabilities for breeding birds that moved between colonies to breed compared to transitions for birds that did not move (Dugger et al., 2010). Therefore, we developed a model structure on Ψ that grouped the remaining 54 transitions not fixed to zero, into categories that reflected general movement behaviors relative to reproductive states that were not colony specific (Table S2). Thus “goers” were birds from any of the 3 breeding colonies that transitioned between reproductive states and moved to a different colony from year *i* to year *i* + 1 and “stayers” were birds from any of the 3 colonies that transitioned between reproductive states but remained at the colony where they were resighted in the previous year (StayGo parameter in models). For example, birds that were pre‐breeders at Royds in year *i* and transitioned not only into the breeding population in year *i* + 1 but also moved to a different colony (either Bird or Crozier) were considered “goers” (${\hat{\Psi }}_{\mathrm{PR}\;\text{to BRgoer}}$). For both “stayers” and “goers” we estimated annual recruitment as the probability of transitioning from the pre‐breeder state to the breeder state (${\hat{\Psi }}_{\mathrm{PR}\;\text{to}\;\mathrm{BR}}$) and breeding propensity was estimated with the annual probability of remaining in the breeding state from 1 year to the next (${\hat{\Psi }}_{\mathrm{BR}\;\text{to}\;\mathrm{BR}}$ or $1-{\hat{\Psi }}_{\mathrm{BR}\;\text{to}\;\mathrm{NR}}$).

It is important to note that because sex was not always assigned the first time a bird was observed, or always with certainty over multiple sightings (within or between years), “cumulative” sightings were used to assign sex. For this reason, the encounter history format we used was expected to generate positively biased survival estimates because most individuals were seen over multiple years before they were sexed, and the more often they were resighted the more likely they were to have sex assigned correctly (e.g., Nichols et al., 2004). However, our objectives were to understand whether probabilities of detection ($\hat{p}$), apparent survival ($\hat{S}$), recruitment, and breeding propensity based on transition probabilities between reproductive states (${\hat{\Psi }}_{\mathrm{PR}\;\text{to}\;\mathrm{BR}}$ and ${\hat{\Psi }}_{\mathrm{BR}\;\text{to}\;\mathrm{BR}}$) varied relative to sex, rather than produce unbiased survival estimates. We believe the approach we used allowed us to generate precise model coefficients for the sex effects on model parameters, thereby detecting sex‐related variation if it existed.

Finally, because no band resighting surveys were performed at Cape Bird after 2013, we coded encounter histories as “dots” (.) during 2014–2019 to denote years when surveys were not conducted, and we did this for all birds banded at Cape Bird colony or last resighted at Cape Bird colony and not resighted subsequently at capes Crozier or Royds. We also coded a sample of birds (*n* = 239) at capes Crozier and Royds during 2016–2018 with “dots” to remove them from the likelihood during the time period when they were wearing geolocator tags on their legs to account for the possibility that these devices might have an added effect on survival or detection probabilities.

We used an information theoretic approach (Burnham & Anderson, 2002) to generate a priori model sets to evaluate specific hypotheses. To identify models with the most support, we used model selection criteria including the corrected version of Akaike's information criterion for small sample sizes (AIC_c_) when including the overdispersion factor, the difference in AIC_c_ between each candidate model and the model with the lowest AIC_c_ value (ΔAIC_c_), and Akaike weights (Burnham & Anderson, 2002). We used Program MARK to generate model estimates and model selection results (White et al., 2006), and we evaluated the strength of support for model coefficients (Betas: $\hat{\beta }$) in competitive models by considering the degree to which 95% confidence limits (CIs) overlapped zero. Coefficients with confidence limits that did not overlap zero were considered to have the strongest support, those with CI's overlapping zero <10% were considered weakly supported and those with CI's broadly overlapping zero were considered to have no support (e.g., Dugger et al., 2016).

To minimize the number of models in the final model set, we generated a priori model sets independently for each parameter (S,p,Ψ) to evaluate predicted responses of Adelie penguins in relation to general time variation (*t*), differences by sex (SEX), breeding colony (for *S* and *p* only; *B*, Bird; *C*, Crozier; COL, all colonies different; *R*, Royds), and reproductive state (BR, breeder; NB, non‐breeder; PR, pre‐breeder; RS, all reproductive states different) (Tables S3–S5). We determined the best structure for recapture, transition, and survival parameters by implementing a combination of a “build‐up” approach within a “secondary candidate set” model development strategy following Morin et al. (2020). We fit sub‐models independently for each parameter and then combined the best model structures for each parameter into a final model set. Within the submodeling stage for *S* and *p*, we built model complexity starting with alternative structures for reproductive state and colony effects, then we incorporated time and sex building on the best “base” models from each previous step (Tables S3 and S4). Transitions between reproductive states were modeled relative to whether a bird changed reproductive states and stayed at the original colony (StayGo; Table S5).

Highly parameterized multistate models can result in unidentifiable, or imprecise parameters (White et al., 2006), thus we were very careful about including interactions in our model structures. We did not expect time to affect either detection rates or survival differently by colony, as presumably environmental factors during the breeding season that might cause variation in *p* or *S* (reflected as temporal variation) were experienced by all the birds in our study, regardless of breeding colony. In addition, time was confounded with reproductive state during the early years of the study. Thus, while we have some evidence that birds from Royds may exhibit post‐breeding season movements that differ from birds that breed at Cape Crozier (Ballard et al., 2010), we opted to include time as an additive effect in order to more precisely estimate sex effects. For similar reasons, we only evaluated the interaction between best reproductive state structure and sex on *S* (i.e., no sex by time interaction) and the interaction between StayGo and sex on Ψ (Tables S3, S5).

During the submodeling process, we maintained a general structure on the nonmodeled parameters that included the additive effects of colony (COL), reproductive state (RS), and general time variation (*t*) for *S* and *p* and for Ψ, the general structure was StayGo + *t*. Models with AIC_c_ < 5 for each parameter from the submodeling stage were retained and then combined in a final model set (i.e., Secondary Candidate Set selection following Morin et al., 2020). We used ∆AIC_c_ ≤ 2 to identify competitive models and draw inference from the final model set.

### 2.2 Adult sex ratio

From the dataset including only known‐sex birds returning as pre‐breeders, breeders, or non‐breeders each year (*n* = 2127), we calculated the annual, adult sex ratio following Ancona et al. (2017), where ASR is estimated as the male proportion of the total population as follows:

$$\mathrm{ASR}=\frac{N_{m}}{N_{m}+N_{f}}$$

with *N*_m_, the number of males observed and *N*_f_, the numbers of females observed. Adélie penguins are rarely observed at natal breeding colonies before age 2, and the average age‐at‐first reproduction is 5.4 at Cape Crozier and 6.0 at Royds and Bird (Kappes et al., 2021). Therefore, we considered our counts of males and females as ASR (ratio of males to females who have reached reproductive age—includes pre‐breeders, breeders, and non‐breeders) rather than operational sex ratio (OSR; ratio of breeding males to breeding females; Ancona et al., 2017). Banded populations were small and band search effort was variable during the first years of the study, particularly at Cape Bird where <10 banded birds were observed each year until 2002. In addition, temporal estimates of detection rates from multistate models can be estimated for time 2 through time *k*, because time 2 (i.e., 1999) is the first year banded birds encountered in the initial year of the study (1998) can be resighted. This meant we calculated ASR at capes Royds and Crozier from 1999 to 2019 and Cape Bird from 2002 to 2013.

Resighting probabilities were high, but <1.0 and varied by sex, so we used sex‐ and colony‐specific annual estimates of detection rates $(p\left(\mathrm{SEX}+\text{Colony}+\text{time})\right)$ for males and females each year. We then estimated abundance for each sex, colony, and year while adjusting for variation in detection rates (Williams et al., 2002):

$${\hat{N}}_{\mathrm{ic}}=\frac{n_{\mathrm{ic}}}{p_{\mathrm{ic}}}$$

In this formula, *N*_ic_ is the estimated sex‐specific abundance at year *i* and colony *c*, *n*_ic_ is the sex‐specific total number of individuals resighted in year *i* at colony *c*, and *p*_ic_ is the estimated sex‐specific resighting probability (probability that a member of *N*_i_ is caught at time *i* at colony *c*) from our survival analysis. Once we calculated sex‐specific annual estimates of abundance for each colony and year, we evaluated differences in the proportion of males and females each year at each colony using a generalized linear model in R (R Core Team, 2020). To avoid violating assumptions associated with a binomial likelihood, we used a quasi‐binomial distribution model (i.e., pseudo‐logistic regression) to determine the additive effects of colony and year on the two‐column integer matrix of adjusted annual counts of males and females, with males denoting the number of successes (Shoukri & Aleid, 2022). We then generated the estimated marginal means (i.e., least squares means) for the proportion of males at each colony and across the entire time series using R package *emmeans* (version 1.7.4.1). Estimates above 0.50 reflected male bias and estimates below 0.50 reflected female bias. If the corresponding (unadjusted) 95% confidence intervals calculated for each estimated probability did not include 0.50, we considered this strong evidence of a skewed ASR for that colony and year.

## 3 RESULTS

Sex was assigned correctly based on visual observations for 97.14% (two‐sided exact binomial test, 95% CI: 0.93 to 0.99) of the birds with both visual and molecular DNA sex determinations (*N* = 140). Thus, we concluded that sex determined by observation was reliable and we used all 2127 birds for which we had sex assignments in the subsequent analyses.

First, we modeled the probability of detection and there was only a single model structure on *p* with ΔAIC_c_ ≤ 5 (Table S3). That model contained the additive influence of colony (Colony), reproductive state (RS), sex (SEX), and time (*t*) $\left[p\left(\text{Colony}+\mathrm{RS}+\mathrm{SEX}+t\right);{\mathrm{AIC}}_{c}\;\mathrm{Wt}.=0.97\right]$. Average annual resighting probabilities were lower at Bird (${\hat{\beta }}_{\text{Bird}}=-0.93,\mathrm{SE}=0.29,95\%\mathrm{CI}:-1.50\;\text{to}-0.35$) and Crozier (${\hat{\beta }}_{\text{Crozier}}=-0.98,\mathrm{SE}=0.19;95\%\mathrm{CI}:-1.36\;\text{to}-0.60$) compared to Royds, and higher for males (${\hat{\beta }}_{\text{male}}=0.39,\mathrm{SE}=0.10;95\%\mathrm{CI}:0.18\;\text{to}\;0.58$) compared to females. Average resighting rates were higher for pre‐breeders relative to non‐breeders (${\hat{\beta }}_{\mathrm{pre}-\text{breeder}}=0.63,\mathrm{SE}=0.11;95\%\mathrm{CI}:0.42\;\text{to}\;0.85$). Precision of the model coefficient for breeders relative to non‐breeders was poor (${\hat{\beta }}_{\text{breeder}}=15.5,\mathrm{SE}=154.6;95\%\mathrm{CI}:-287.54\;\text{to}\;318.62$), likely because resighting rates for breeders were estimated to be between 0.99 and 1.0 in all years. Mean resighting rates across both sexes for pre‐breeders ranged from lows in 2007 of 0.77 (SE = 0.03, 95% CI: 0.70 to 0.83) at Cape Crozier and highs in 2009 of 0.98 (SE = 0.006, 95% CI: 0.96 to 0.99) at Cape Royds. Mean resighting rates across both sexes for non‐breeders ranged from lows in 2007 of 0.64 (SE = 0.04, 95% CI: 0.55 to 0.72) at Cape Crozier, and highs in 2009 of 0.96 (SE = 0.01, 95% CI: 0.93 to 0.98) at Cape Royds. Estimates for Cape Bird fell between the highs and lows at capes Royds and Crozier. This model structure on p $\left[\left(\text{Colony}+\mathrm{RS}+\mathrm{SEX}+t\right)\right]$ was the only one carried forward in the modeling process.

Only two models during the submodeling stage for Ψ had a ΔAIC_c_ ≤ 5 and both models included a sex effect (Table S4). Six model structures for apparent survival were competitive (∆AIC_c_ ≤ 5) and all 6 models contained the additive effects of colony, reproductive state, and time and 3 models also included either the additive effect of sex, or the interaction between sex and reproductive state (Table S5).

The final model set included combinations of the best structures on *S*, *p* and Ψ from the submodeling stages and 3 models were competitive (≤2 AIC_c_) and comprised 65% of the AIC_c_ weight (Table 1). All competitive models included the interaction between sex and transitions between reproductive states (i.e., “stayers” vs. “goers”; StayGo), and the additive effect of time $\left[\Psi \left(\text{StayGo}*\mathrm{SEX}+t\right)\right]$, as well as the only structure selected for *p* from the submodeling stage $\left[p\left(\text{Colony}+\mathrm{RS}+\mathrm{SEX}+t\right)\right]$.

**TABLE 1:** Model selection results for the final multistate mark recapture model set estimating apparent survival (S), the probability of resighting (p), and transition probabilities (Ψ) relative to general time variation, breeding colony, and reproductive state for Adélie penguins from 1998 to 2019.

Model^a^	∆AIC_c_ ^b^	*K* ^c^	*w*_*i*_ ^d^	Deviance^e^
$S\left(\mathrm{COL}+\mathrm{PR},\mathrm{BR}=\mathrm{NB}+t\right)\;p\left(\text{best}\right)\Psi \left(\text{StayGo}*\mathrm{SEX}+t\right)$	0.000	87	0.334	24317.55
$S\left(\mathrm{COL}+\mathrm{PR},\mathrm{BR}=\mathrm{NB}+\mathrm{SEX}+t\right)\;p\left(\text{best}\right)\Psi \left(\text{StayGo}*\mathrm{SEX}+t\right)$	1.397	88	0.166	24316.92
$S\left(\mathrm{COL}+\mathrm{RS}+t\right)\;p\left(\text{best}\right)\;\Psi \left(\text{StayGo}*\mathrm{SEX}+t\right)$	1.577	88	0.152	24317.10
$S\left(\mathrm{COL}+\mathrm{RS}+\mathrm{SEX}+t\right)\;p\left(\text{best}\right)\;\Psi \left(\text{StayGo}*\mathrm{SEX}+t\right)$	2.955	89	0.076	24316.44
$S\left(\mathrm{COL}+\mathrm{PR},\mathrm{BR}=\mathrm{NB}*\mathrm{SEX}+t\right)\;p\left(\text{best}\right)\;\Psi \left(\text{StayGo}*\mathrm{SEX}+t\right)$	3.112	89	0.070	24316.60
$S\left(\mathrm{COL}+\mathrm{PR},\mathrm{BR}=\mathrm{NB}+t\right)\;p\left(\text{best}\right)\;\Psi \left(\text{StayGo}+\mathrm{SEX}+t\right)$	3.263	79	0.065	24337.07
$S\left(B,R=C+\mathrm{PR},\mathrm{BR}=\mathrm{NB}+t\right)\;p\left(\text{best}\right)\;\Psi \left(\text{StayGo}*\mathrm{SEX}+t\right)$	4.269	86	0.039	24323.86
$S\left(\mathrm{COL}+\mathrm{PR},\mathrm{BR}=\mathrm{NB}+\mathrm{SEX}+t\right)\;p\left(\text{best}\right)\;\Psi \left(\text{StayGo}+\mathrm{SEX}+t\right)$	4.703	80	0.032	24336.48
$S\left(\mathrm{COL}+\mathrm{RS}+t\right)\;p\left(\text{best}\right)\;\Psi \left(\text{StayGo}+\mathrm{SEX}+t\right)$	4.859	80	0.029	24336.63
$S\left(\mathrm{COL}+\mathrm{RS}+\mathrm{SEX}+t\right)\;p\left(\text{best}\right)\;\Psi \left(\text{StayGo}+\mathrm{SEX}+t\right)$	6.280	81	0.014	24336.02

*Note*: Movement between reproductive states was modeled as differences between individuals that stay at their current colony vs. those that move to another colony for the transition (StayGo).

^a^ *B*, Bird; BR, breeder; *C*, Crozier; COL, all colonies different; NB, non‐breeder; PR, pre‐breeder; *R*, Royds; RS, all reproductive states different; SEX, binary covariate with males = 1, females = 0; t, general time variation; All models included the best structure from the submodeling stage for $p\;\left[p\left(\text{best}\right)=p\left(\mathrm{COL}+\mathrm{RS}+\mathrm{SEX}+t\right)\right]$.

^b^ Differences between Akaike's Information Criteria adjusted for small sample size (AIC_c_) and AIC_c_ from the top model (ΔAIC_c_) with AIC_c_ from top model = 24492.98.

^c^ Number of model parameters (*K*).

^d^ AIC_c_ weights (*w*_i_).

^e^ Model deviance.

**FIGURE 1:**
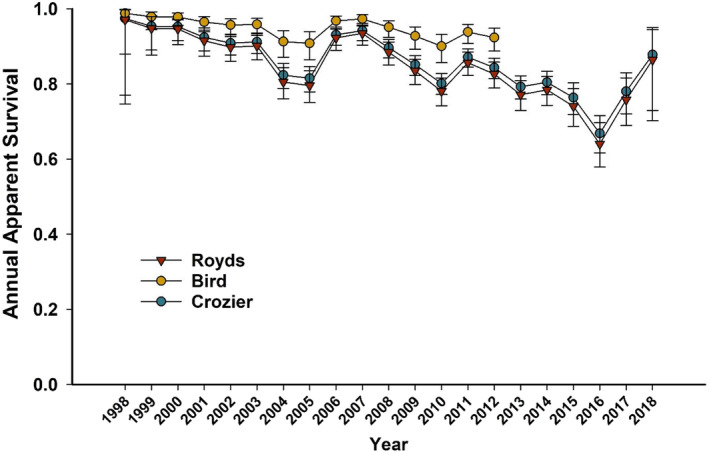
Mean annual estimates and 95% confidence limits of apparent survival for Adélie penguins at 3 breeding colonies on Ross Island, Antarctica from 1998 to 2019 for capes Royds and Crozier, and 1998–2013 for Cape Bird. Estimates reflect annual means across all reproductive states (RS) for each colony from model $\left[S\left(\text{Colony}+\text{time}\right)\;p\left(\text{Colony}+\mathrm{RS}+\mathrm{SEX}+\text{time}\right)\Psi \;\left(\text{StayGo}*\mathrm{SEX}+\text{time}\right)\right]$.

**FIGURE 2:**
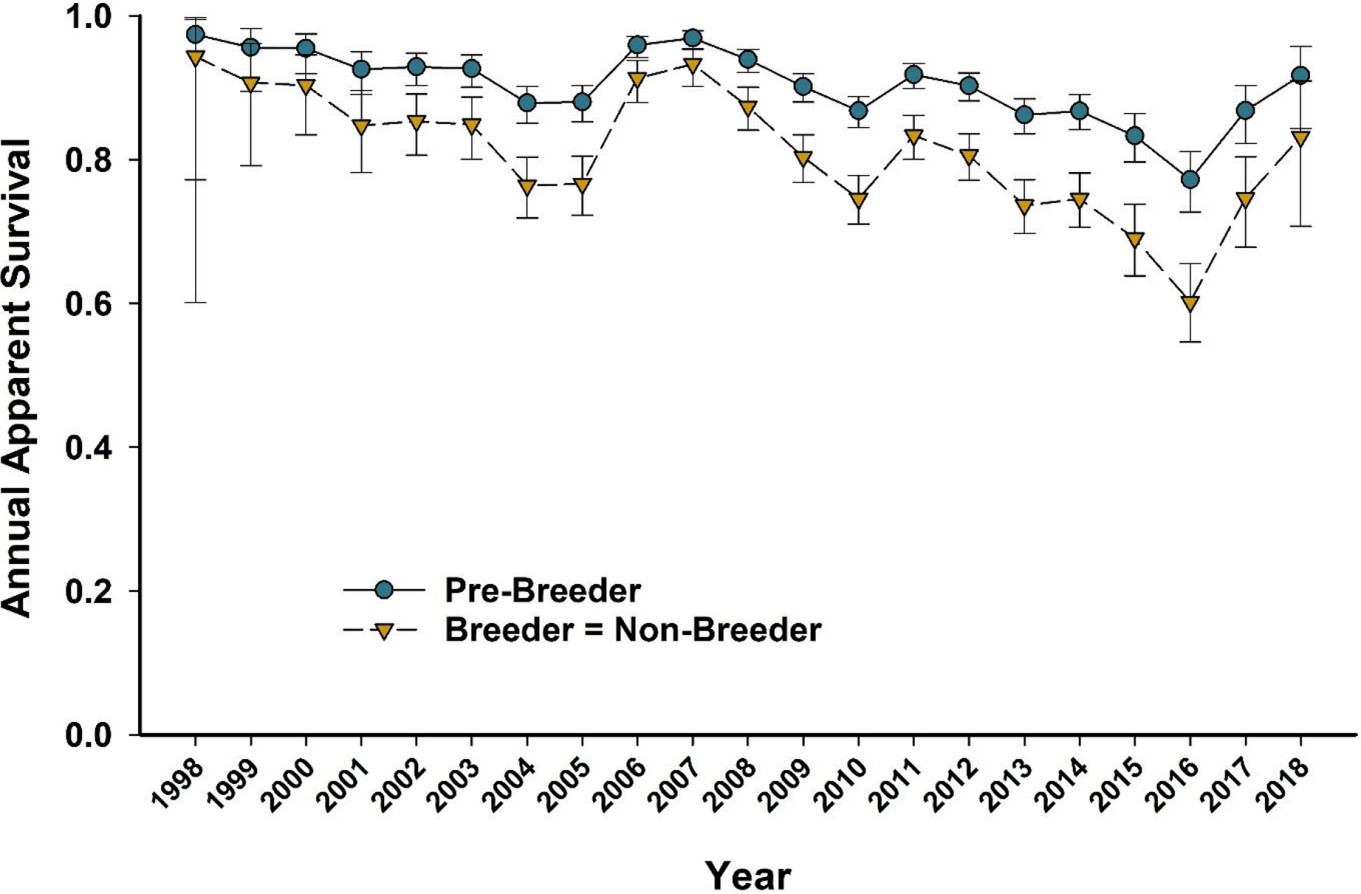
Mean annual estimates and 95% confidence limits for apparent survival relative to reproductive state for Adélie penguins at three breeding colonies on Ross Island, Antarctica from 1998 to 2019 for capes Royds and Crozier, and 1998–2013 for Cape Bird. Estimates reflect annual means across all colonies for each reproductive state from model $S\left[\left(\mathrm{PR},\mathrm{BR}=\mathrm{NB}+\text{time}\right)p\left(\text{Colony}+\mathrm{RS}+\mathrm{SEX}+\text{time}\right)\Psi \left(\text{StayGo}*\mathrm{SEX}+\text{time}\right)\right]$.

Survival differences by colony were strongly supported with the highest apparent survival rates observed at Bird (${\hat{\beta }}_{\text{Bird}}=0.95,\mathrm{SE}=0.21;95\%\mathrm{CI}:0.53\;\text{to}\;1.36$), followed by Crozier (${\hat{\beta }}_{\text{Crozier}}=0.20,\mathrm{SE}=0.08;95\%\mathrm{CI}:0.05\;\text{to}\;0.35$), which had survival rates only slightly higher than Royds (Figure 1). The top 2 models represented 52% of the AIC_c_ weight and strongly supported higher rates of survival for pre‐breeders relative to breeders and non‐breeders, which were similar (${\hat{\beta }}_{\mathrm{pre}-\text{breeder}}=0.81,\mathrm{SE}=0.06;95\%\mathrm{CI}:0.69\;\text{to}\;0.93$) (Figure 2). The top‐ranked model did not include the influence of sex on apparent survival and had 2.01 times more support than the 2nd best model that included a sex effect (Table 1). In addition, the model coefficient for sex in the 2nd ranked model was small and imprecise with 95% CIs that widely overlapped zero (${\hat{\beta }}_{\mathrm{sex}}=0.04,\mathrm{SE}=0.06;95\%\mathrm{CI}:-0.07\;\text{to}\;0.15$), suggesting little support for a sex effect on survival.

**FIGURE 3:**
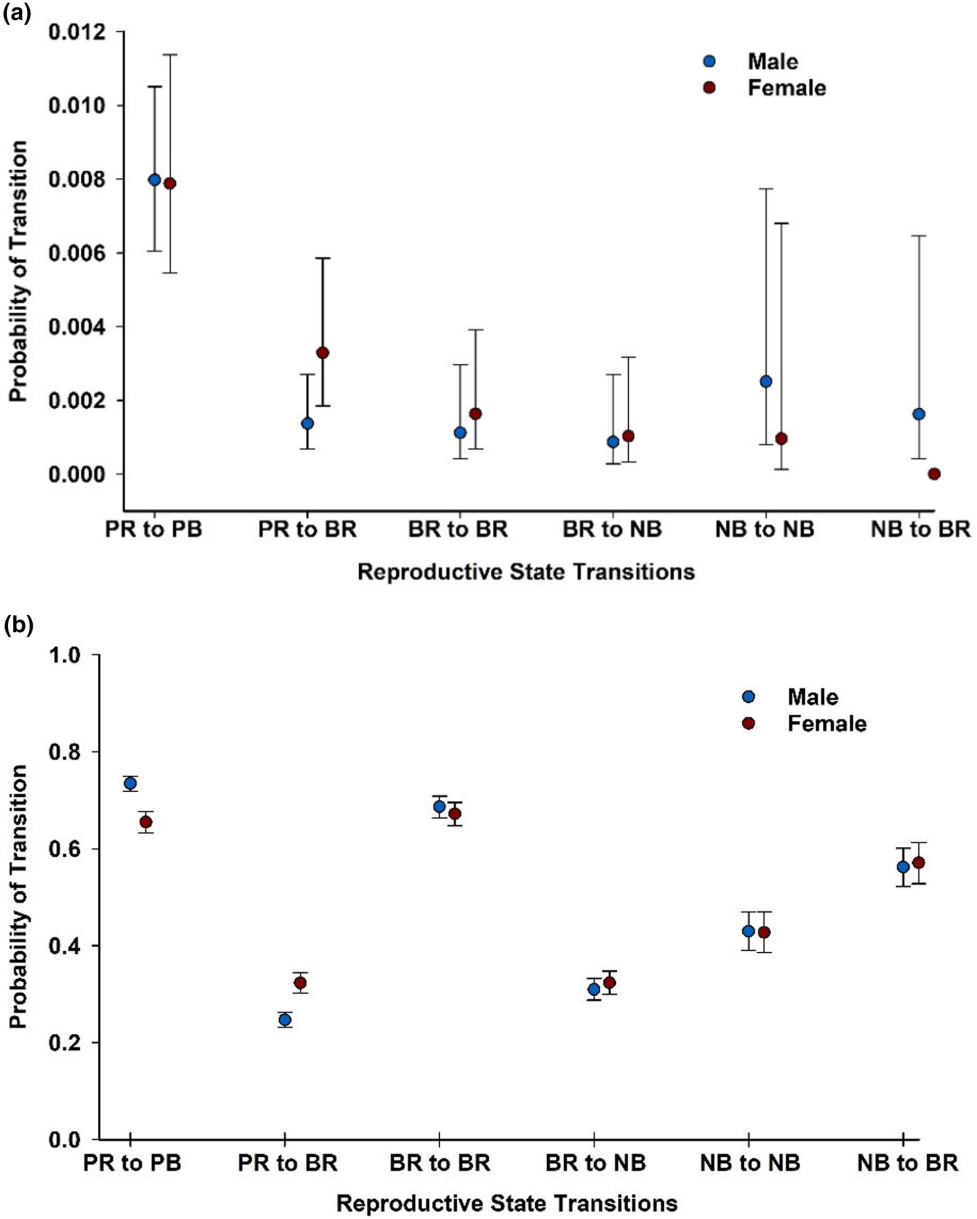
The mean probability of transitioning ($\hat{\Psi }$) between reproductive stages (BR, Breeding; NB, Non‐breeding; PR, pre‐breeding) with 95% Confidence limits for male and female Adélie penguins at 3 breeding colonies (capes Royds, Crozier, Bird) on Ross Island, Antarctica, 1998–2019 for birds that (a) moved to another colony before the transition (“goers”), and (b) remained at the same colony after changing reproductive states (“stayers”). Estimates of Ψ are averaged over time from model $S\left(\text{Colony}+\mathrm{PR},\mathrm{BR}=\mathrm{NB}+\text{time}\right)\;p\left(\text{Colony}+\mathrm{RS}+\mathrm{SEX}+\text{time}\right)\;\Psi \;\left(\text{StayGo}*\mathrm{SEX}\right)$.

Across the entire time series, the average probability of transitioning between breeding states and moving to a new colony was very low (<1% for all transitions; Figure 3a), relative to the probability of changing breeding states and staying at the colony of origin (Figure 3b). Interestingly, the average probability of recruiting into the breeding population across all colonies (i.e., $\Psi_{\mathrm{Pre}-\text{Breeder to Breeder}}$) was higher for females than for males (${\hat{\beta }}_{\text{Male}}=-0.39,\mathrm{SE}=0.07;95\%\mathrm{CI}:-0.52\;\text{to}-0.26$) for birds who moved before breeding as well as for those who stayed (Figure 3a,b). However, regardless of movement behavior, breeding propensity or the probability that a bird remains a breeder in the following year ($\Psi_{\text{Breeder to Breeder}}$) did not vary between the sexes (Figure 3a,b). There is some indication that males that take a breeding sabbatical are more likely to move the subsequent year and either remain a non‐breeder ($\Psi_{\text{Non}-\text{Breeder to Non}-\text{Breeder}}$), or return to breeding ($\Psi_{\text{Non}-\text{Breeder to Breeder}}$) relative to females, but overall, movement of birds once they begin breeding occurs very infrequently (Figure 3a).

**FIGURE 4:**
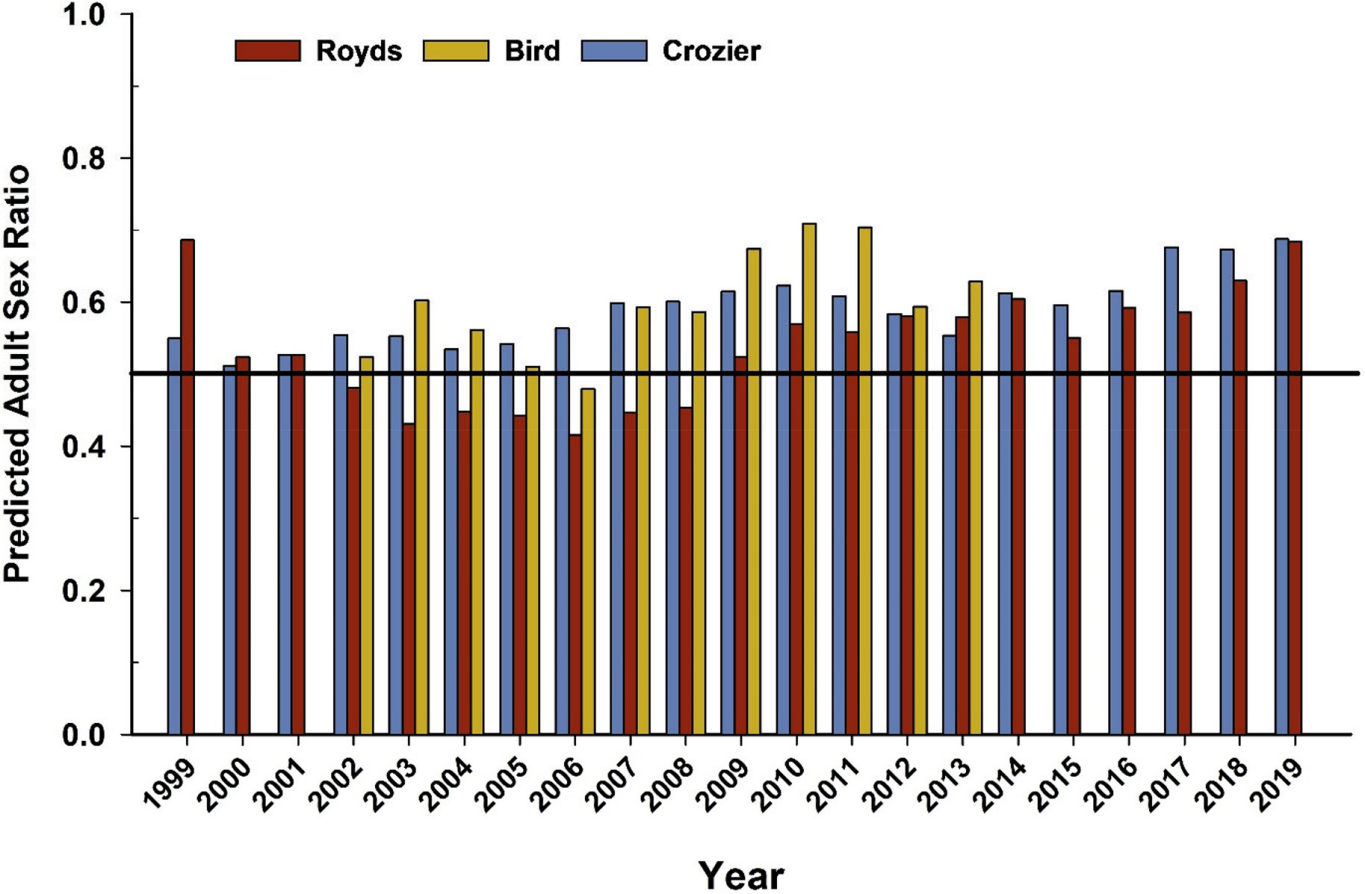
Annual predicted estimates of adult sex ratio (ASR) calculated for Adélie penguins at three breeding colonies on Ross Island, Antarctica from 1999 to 2019 for capes Royds and Crozier, and 2002–2013 for Cape Bird.

After correcting the annual counts of banded birds observed at each colony by sex‐, colony‐, and annual resighting probabilities, we found that mean ASR across all years and colonies was strongly male‐biased (1.4:1 total adjusted counts; $\hat{x}$ proportion of males = 0.57, SD = 0.07). Mean ASR across all years by colony ranged from 0.61 (SE=0.03,95%CI:0.55to0.67) at Cape Bird, to 0.59 (SE=0.01,95%CI:0.58to0.61) at Cape Crozier, and 0.53 (SE=0.02,95%CI:0.50to0.57) at Cape Royds. Annual patterns in ASR illustrated significant male‐bias over almost all years at all colonies across the entire study period, with the exception at Cape Royds from 2002 to 2008, and Cape Bird during 2006 when ASR was female‐biased (Figure 4).

## 4 DISCUSSION

To our knowledge, this is the first study to use multistate models to evaluate differences in annual survival, recruitment, and breeding propensity relative to sex for a known‐breeding history Adélie penguin metapopulation. Interestingly, we found little evidence that survival varied by sex, but much stronger evidence that annual survival was higher for pre‐breeders (0.90, SE = 0.004, 95% CI: 0.89 to 0.91) compared to breeders and non‐breeders (0.80, SE = 0.006, 95% CI: 0.78 to 0.81). In addition, the probability of recruitment into the breeding population was on average ~7% higher each year for females (0.32, SE = 0.011, 95% CI: 0.30 to 0.35) relative to males (0.24, SE = 0.008, 95% CI: 0.23 to 0.26). Earlier recruitment coupled with lower survival once birds become breeders, would suggest that females in this metapopulation have shorter lifespans relative to males, findings consistent with an earlier study conducted at Cape Crozier in the late 1970s (Ainley et al., 1983; Ainley & DeMaster, 1980). We can apply mean differences in recruitment rates and survival observed in this study between pre‐breeders and breeders to a hypothetical population of 2000, 2‐year‐old pre‐breeders with an unbiased sex ratio (i.e., ASR = 0.50; 1000 males, 1000 females). Given the mean vital rates estimated in this study, by approximately age 9, when pre‐breeder survival begins to decline in this population (KMD, GB, DGA unpublished data), the sex ratio would be strongly male‐skewed (ASR = 0.65) with 8.8% of the original male cohort still alive, versus 4.5% of the female cohort (Tables S6 and S7). Thus, sex‐related differences in survival may not be the only mechanism that can drive biased sex‐ratios (e.g., Gownaris & Boersma, 2019) and differences in recruitment rates between the sexes and survival relative to breeding state may contribute to the largely male‐biased sex ratios for Adélie penguins that we observed at all colonies during most years.

Sexual size dimorphism in many penguin species is relatively small but in contrast to our study, decreased survival for females has been reported for African penguins (*Spheniscus demersus*; Spelt & Pichegru, 2017), Magellanic penguins (*Spheniscus magellanicus*; Vanstreels et al., 2013, Gownaris & Boersma, 2019), King penguins (*Aptenodytes patagonicus*; Olsson & Van der Jeugd, 2002), and Adélie penguins (Ainley & DeMaster, 1980), although this early study did not disentangle sex‐related vital rate differences relative to breeding state, recruitment, or breeding propensity. Consistent with findings for the Cape Crozier population from earlier in the current time series (Dugger et al., 2006), annual survival in our study did not differ significantly between breeding males and females, despite differences in size and foraging behavior at the largest colonies due to increased levels of intraspecific trophic competition (cf Lescroël et al., 2014, 2020; Saenz et al., 2020).

Variation in age‐at‐first breeding or recruitment rate by sex, with females breeding at a younger age or with a higher annual probability than males, has been reported for the Common tern (*Sterna hirundo*; Becker et al., 2008), Nazca Booby (*Sula granti*; Tompkins & Anderson, 2019) and Wandering albatross (*Diomedea exulans*; Fay et al., 2015), presumably because skewed sex ratios decreased mate availability for the more common sex. However, under this scenario other mechanisms (e.g., sex‐related survival differences) at some stage of the species' life history must be responsible for biased sex ratio at recruitment age. While sex‐related differences in adult survival are most often identified as the primary cause of biased ASRs in birds (Székely, Weissing, & Komdeur, 2014), there are a variety of other potential mechanisms that can contribute to observed patterns in ASR including (1) sex‐biased primary sex ratios occurring at hatch or during chick‐rearing, (2) sex‐related differences in subadult or juvenile survival, or (3) sex‐biased dispersal or permanent emigration during either juvenile or adult life stages.

Sex ratio at hatching is not well‐studied in Adélie penguins, but no sex ratio differences at hatching were observed relative to hatching order, brood size, or between study years at Cape Crozier during 2012–2013 (Jennings et al., 2016), and apparent daily survival rates of chicks did not vary relative to sex (Jennings et al., 2023). Survival differences in Adélie penguins could arise after fledging (i.e., juvenile stage), but they do not appear to be present during chick‐rearing, although male chicks grow faster than female chicks (Jennings et al., 2016, 2021).

Small sex‐related differences in vital rates, including subadult or juvenile survival can accumulate into relatively large biases in ASR (e.g., Gownaris & Boersma, 2019). Increased female mortality during the juvenile stage has been reported for Magellanic penguins and the resulting male‐biased juvenile survival made the greatest contribution to population declines and increased ASR biases over a 20‐year time series (Gownaris & Boersma, 2019). Similarly, male‐biased juvenile survival made the highest contribution to the skewed ASR observed in green‐rumped parrotlets (*Forpus passerinus*), whereas differences in the cost of reproduction between sexes only played an intermediate role (Veran & Beissinger, 2009). We did not sex chicks at banding (required DNA analysis), so we could not evaluate sex‐related variation in survival during the first 2 years post‐fledging, but survival of fledglings (fledging to age 1) can be low and highly variable among years (Emmerson & Southwell, 2011; Hostetler et al., 2021). Although no information exists regarding sex‐related differences in Adélie penguin survival during early life, it is possible such a variation could occur and make a significant contribution to the male‐biased sex‐ratio in our Adelie penguin metapopulation. Thus, although differences in recruitment rates, and decreased survival of both male and female breeders are contributing to the male‐biased sex ratios observed in our study, we cannot discount the potential contribution of sex‐related survival differences for Adélie penguins during the first few years of life.

Finally, while rates of permanent emigration away from natal subcolonies during the unobservable subadult stage are difficult to measure for many seabirds (e.g., Coulson, 2016), we found movement rates between colonies in our metapopulation to be episodic and quite small relative to pre‐breeders who remained pre‐breeders at their current colony (Figure 3b; LaRue et al., 2013). Thus, it seems unlikely that such movements would consistently carry females out of our 4‐colony metapopulation (i.e., permanent emigration) at a higher rate than males even before they begin to breed.

While mean ASR was generally male biased in our study (0.57, SD = 0.07), we observed some interesting annual patterns in colony‐specific ASR during 2002–2008, the only part of the time series in which ASR was female‐biased at Cape Royds, and during 2006 at Cape Bird (Figure 4). Factoring in a 2‐year time lag, this time period coincided with persistent, extensive sea ice in the region, especially for the Cape Royds and Cape Bird colonies (Dugger et al., 2014). The extensive sea ice resulted in long walks to and from open water for foraging, in some cases throughout most of chick‐rearing at Royds (Dugger et al., 2014). These long walks to reach foraging habitats increased the amount of time males had to fast during the first incubation stint while females were away regaining body condition after egg laying (Ainley, 2002) and, with nest desertions by waiting partners, decreased overall breeding success between 2000 and 2005 (Dugger et al., 2014). The additional energetic expense of foraging during these breeding seasons may have increased male mortality or permanent emigration relative to females during those years. However, if so, such a survival impact was not strong enough across the 22‐year time series to result in survival differences between males and females in our model. Movement of breeding birds away from the Cape Royds colony was higher during the years of extensive sea ice (2000–2005) for both sexes (Dugger et al., 2010), but still very low and comparable to movement rates for “goers” estimated from this longer study. In combination, small survival differences, or increased movement behavior resulting in permanent emigration by males might have decreased the male bias at Cape Royds, in particular.

Understanding variation in population‐level vital rates is needed to understand underlying mechanisms driving population dynamics and trajectories. Here, we document sex‐ and breeding status‐related variation in recruitment rates, which in combination, likely contribute to the male‐biased ASR observed in this Adélie penguin metapopulation. Male‐biased ASR is common in many wild bird taxa (Donald, 2007; Székely, Weissing, & Komdeur, 2014), but increased imbalances between males and females have been associated with small, disappearing colonies/populations (Donald, 2007). Thus, the genetic and demographic consequences of skewed sex ratios may have conservation implications for some species. When sex‐related survival differences are important characteristics of population dynamics, increased bias in ASR can reflect important survival changes that might be expected to result in population declines (e.g., Gownaris & Boersma, 2019). However, consistent with a more complicated mechanism involving recruitment rates and survival differences by breeding state, we observed increasing population trajectories at three of the four colonies we studied (including Beaufort; Lyver et al., 2014). Thus, current levels of male‐biased ASR do not appear to be associated with declining populations in our metapopulation, although the factors behind the slow recovery at Cape Royds after an environmental disturbance (Dugger et al., 2014) are still under investigation (Schmidt et al., 2021).

## AUTHOR CONTRIBUTIONS

**Virginia Morandini:** Conceptualization (equal); data curation (lead); formal analysis (lead); methodology (equal); writing – original draft (lead); writing – review and editing (supporting). **Katie M. Dugger:** Conceptualization (equal); data curation (equal); formal analysis (supporting); funding acquisition (equal); investigation (equal); methodology (equal); project administration (lead); supervision (lead); writing – original draft (supporting); writing – review and editing (equal). **Annie E. Schmidt:** Data curation (supporting); funding acquisition (supporting); writing – original draft (supporting); writing – review and editing (supporting). **Arvind Varsani:** Formal analysis (supporting); resources (supporting); writing – original draft (supporting); writing – review and editing (supporting). **Amélie Lescroël:** Data curation (supporting); funding acquisition (supporting); writing – original draft (supporting); writing – review and editing (supporting). **Grant Ballard:** Conceptualization (supporting); data curation (equal); funding acquisition (equal); investigation (supporting); methodology (supporting); project administration (equal); resources (equal); supervision (supporting); writing – original draft (supporting); writing – review and editing (supporting). **Phil O'B. Lyver:** Conceptualization (supporting); data curation (equal); funding acquisition (equal); methodology (equal); project administration (equal); resources (equal); writing – original draft (supporting); writing – review and editing (supporting). **Kerry Barton:** Conceptualization (supporting); data curation (equal); methodology (supporting); writing – original draft (supporting); writing – review and editing (supporting). **David G. Ainley:** Conceptualization (equal); data curation (equal); funding acquisition (equal); project administration (equal); writing – original draft (supporting); writing – review and editing (supporting).

## CONFLICT OF INTEREST STATEMENT

None.

## STATEMENT ON INCLUSION

Our study brings together authors from four different countries that are signatories on the Antarctic Treaty. This includes scientists based in the two countries that supported the field logistics at all our study sites on Ross Island, Antarctica. All authors were engaged early on with the research and study design to ensure that the diverse sets of perspectives they represent were considered from the onset. Whenever relevant, literature published by scientists who have conducted research in the region was cited.

## Supporting information


Appendix S1.

## Data Availability

Data collected and used fro this research are available from California Avian Data Center (CADC) hosted by Point Blue Conservation Science (http://data.pointblue.org/apps/penguinscience/) and are also available from the US Antarctic Program Data Center (https://www.usap‐dc.org/view/project/p0010179).
