# Supplementary material for: Sex‐specific recruitment rates contribute to male‐biased sex ratio in Adélie penguins
Source: Ecol Evol. 2024 Feb 20;14(2):e10859. doi: 10.1002/ece3.10859 (PMC10879839; doi:10.1002/ece3.10859)
Supplement: Supplementary file 1 — Appendix S1. [file ECE3-14-e10859-s001.docx]

**SUPPLEMENTARY MATERIAL**

**A. Multi-state model transition probabilities (**$\boldsymbol{\Psi}$**)**

Table S1. All possible transition probabilities ($\Psi_{state in year i to state in year i+1}$) from multi-state mark recapture model with 9 states representing transitions between 3 reproductive states (Pre-breeder = PR (a), Breeder = BR (b), and Non-breeder = NB(c)) and movements between 3 colonies (Royds=R, Bird=B, Crozier=C) from *year i* to *year i+1*. Birds could transition between breeding and non-breeding states while moving between colonies, but pre-breeders could only remain pre-breeders or recruit into the breeding population, so 27 transitions (in bold, columns 3, 4, 7) were not possible and fixed to zero. Transition probabilities for birds that did not move between colonies or change reproductive states were derived through subtraction (denoted by asterisks).

a) Pre-Breeding reproductive state at *year i*

| 1 | 2 | **3** |
| --- | --- | --- |
| $\Psi\left( {PR}_{R}to{PR}_{R} \right)*$ | $\Psi({PR}_{R}to{BR}_{R})$ | $\boldsymbol{\Psi}\boldsymbol{(}\boldsymbol{PR}_{\boldsymbol{R}}\boldsymbol{to}\boldsymbol{NB}_{\boldsymbol{R}}\boldsymbol{)}$ |
| $\Psi({PR}_{R}to{PR}_{B})$ | $\Psi({PR}_{R}to{BR}_{B})$ | $\boldsymbol{\Psi}\boldsymbol{(}\boldsymbol{PR}_{\boldsymbol{R}}\boldsymbol{to}\boldsymbol{NB}_{\boldsymbol{B}}\boldsymbol{)}$ |
| ${\Psi(PR}_{R}to{PR}_{C})$ | $\Psi({PR}_{R}to{BR}_{C})$ | $\boldsymbol{\Psi}\boldsymbol{(}\boldsymbol{PR}_{\boldsymbol{R}}\boldsymbol{to}\boldsymbol{NR}_{\boldsymbol{C}}\boldsymbol{)}$ |
| $\Psi({PR}_{B}to{PR}_{B})*$ | $\Psi({PR}_{B}to{BR}_{B})$ | ${\boldsymbol{\Psi}\boldsymbol{(PR}}_{\boldsymbol{B}}\boldsymbol{to}\boldsymbol{NB}_{\boldsymbol{B}}\boldsymbol{)}$ |
| $\Psi({PR}_{B}to{PR}_{C})$ | $\Psi({PR}_{B}to{BR}_{C})$ | $\boldsymbol{\Psi}\boldsymbol{(}\boldsymbol{PR}_{\boldsymbol{B}}\boldsymbol{to}\boldsymbol{NB}_{\boldsymbol{C}}\boldsymbol{)}$ |
| $\Psi({PR}_{B}to{PR}_{R})$ | $\Psi({PR}_{B}to{BR}_{R)}$ | $\boldsymbol{\Psi}\boldsymbol{(}\boldsymbol{PR}_{\boldsymbol{B}}\boldsymbol{to}\boldsymbol{NB}_{\boldsymbol{R}}\boldsymbol{)}$ |
| $\Psi({PR}_{C}to{PR}_{C})*$ | $\Psi({PR}_{C}to{BR}_{C})$ | $\boldsymbol{\Psi}\boldsymbol{(}\boldsymbol{PR}_{\boldsymbol{C}}\boldsymbol{to}\boldsymbol{NB}_{\boldsymbol{C}}\boldsymbol{)}$ |
| $\Psi({PR}_{C}to{PR}_{B})$ | $\Psi({PR}_{C}to{BR}_{B})$ | $\boldsymbol{\Psi}\boldsymbol{(}\boldsymbol{PR}_{\boldsymbol{C}}\boldsymbol{to}\boldsymbol{NB}_{\boldsymbol{B}}\boldsymbol{)}$ |
| $\Psi({PR}_{C}to{PR}_{R})$ | $\Psi({PR}_{C}to{BR}_{R})$ | $\boldsymbol{\Psi}\boldsymbol{(}\boldsymbol{PR}_{\boldsymbol{C}}\boldsymbol{to}\boldsymbol{NB}_{\boldsymbol{R}}\boldsymbol{)}$ |

b). Breeding reproductive state at *year i*

| **4** | 5 | 6 |
| --- | --- | --- |
| $\boldsymbol{\Psi}\boldsymbol{(}\boldsymbol{BR}_{\boldsymbol{R}}\boldsymbol{to}\boldsymbol{PB}_{\boldsymbol{R}}\boldsymbol{)}$ | $\Psi({BR}_{R}to{BR}_{R}\boldsymbol{)}*$ | $\Psi({BR}_{R}to{NB}_{R}\boldsymbol{)}$ |
| $\boldsymbol{\Psi}\boldsymbol{(}\boldsymbol{BR}_{\boldsymbol{R}}\boldsymbol{to}\boldsymbol{PB}_{\boldsymbol{B}}\boldsymbol{)}$ | $\Psi({BR}_{R}to{BR}_{B}\boldsymbol{)}$ | $\Psi({BR}_{R}to{NB}_{B}\boldsymbol{)}$ |
| $\boldsymbol{\Psi}\boldsymbol{(}\boldsymbol{BR}_{\boldsymbol{R}}\boldsymbol{to}\boldsymbol{PB}_{\boldsymbol{C}}\boldsymbol{)}$ | $\Psi({BR}_{R}to{BR}_{C}\boldsymbol{)}$ | $\Psi({BR}_{R}to{NB}_{C}\boldsymbol{)}$ |
| $\boldsymbol{\Psi}\boldsymbol{(}\boldsymbol{BR}_{\boldsymbol{B}}\boldsymbol{to}\boldsymbol{PB}_{\boldsymbol{B}}\boldsymbol{)}$ | $\Psi({BR}_{B}to{BR}_{B}\boldsymbol{)}*$ | $\Psi({BR}_{B}to{NB}_{B}\boldsymbol{)}$ |
| $\boldsymbol{\Psi}\boldsymbol{(}\boldsymbol{BR}_{\boldsymbol{B}}\boldsymbol{to}\boldsymbol{PB}_{\boldsymbol{C}}\boldsymbol{)}$ | $\Psi({BR}_{B}to{BR}_{C}\boldsymbol{)}$ | $\Psi({BR}_{B}to{NB}_{C}\boldsymbol{)}$ |
| $\boldsymbol{\Psi}\boldsymbol{(}\boldsymbol{BR}_{\boldsymbol{B}}\boldsymbol{to}\boldsymbol{PB}_{\boldsymbol{R}}\boldsymbol{)}$ | $\Psi({BR}_{B}to{BR}_{R}\boldsymbol{)}$ | $\Psi({BR}_{B}to{NB}_{R}\boldsymbol{)}$ |
| $\boldsymbol{\Psi}\boldsymbol{(}\boldsymbol{BR}_{\boldsymbol{C}}\boldsymbol{to}\boldsymbol{PB}_{\boldsymbol{C}}\boldsymbol{)}$ | $\Psi({BR}_{C}to{BR}_{C}\boldsymbol{)}*$ | $\Psi({BR}_{C}to{NB}_{C}\boldsymbol{)}$ |
| $\boldsymbol{\Psi}\boldsymbol{(}\boldsymbol{BR}_{\boldsymbol{C}}\boldsymbol{to}\boldsymbol{PB}_{\boldsymbol{B}}\boldsymbol{)}$ | $\Psi({BR}_{C}to{BR}_{B}\boldsymbol{)}$ | $\Psi({BR}_{C}to{NB}_{B}\boldsymbol{)}$ |
| $\boldsymbol{\Psi}\boldsymbol{(}\boldsymbol{BR}_{\boldsymbol{C}}\boldsymbol{to}\boldsymbol{PB}_{\boldsymbol{R}}\boldsymbol{)}$ | $\Psi({BR}_{C}to{BR}_{R}\boldsymbol{)}$ | $\Psi({BR}_{C}to{NB}_{R}\boldsymbol{)}$ |

c). Non-Breeding reproductive state at *year i*

| **7** | 8 | 9 |
| --- | --- | --- |
| $\boldsymbol{\Psi}\boldsymbol{(}\boldsymbol{NB}_{\boldsymbol{R}}\boldsymbol{to}\boldsymbol{PB}_{\boldsymbol{R}}\boldsymbol{)}$ | $\Psi({NB}_{R}to{BR}_{R}\boldsymbol{)}$ | $\Psi({NB}_{R}to{NB}_{R}\boldsymbol{)}*$ |
| $\boldsymbol{\Psi}\boldsymbol{(}\boldsymbol{NB}_{\boldsymbol{R}}\boldsymbol{to}\boldsymbol{PB}_{\boldsymbol{B}}\boldsymbol{)}$ | $\Psi({NB}_{R}to{BR}_{B}\boldsymbol{)}$ | $\Psi({NB}_{R}to{NB}_{B}\boldsymbol{)}$ |
| $\boldsymbol{\Psi}\boldsymbol{(}\boldsymbol{NB}_{\boldsymbol{R}}\boldsymbol{to}\boldsymbol{PB}_{\boldsymbol{C}}\boldsymbol{)}$ | $\Psi({NB}_{R}to{BR}_{C}\boldsymbol{)}$ | $\Psi({NB}_{R}to{NB}_{C}\boldsymbol{)}$ |
| $\boldsymbol{\Psi}\boldsymbol{(}\boldsymbol{NB}_{\boldsymbol{B}}\boldsymbol{to}\boldsymbol{PB}_{\boldsymbol{B}}\boldsymbol{)}$ | $\Psi({NB}_{B}to{BR}_{B}\boldsymbol{)}$ | $\Psi({NB}_{B}to{NB}_{B}\boldsymbol{)}*$ |
| $\boldsymbol{\Psi}\boldsymbol{(}\boldsymbol{NB}_{\boldsymbol{B}}\boldsymbol{to}\boldsymbol{PB}_{\boldsymbol{C}}\boldsymbol{)}$ | $\Psi({NB}_{B}to{BR}_{C}\boldsymbol{)}$ | $\Psi({NB}_{B}to{NB}_{C}\boldsymbol{)}$ |
| $\boldsymbol{\Psi}\boldsymbol{(}\boldsymbol{NB}_{\boldsymbol{B}}\boldsymbol{to}\boldsymbol{PB}_{\boldsymbol{R}}\boldsymbol{)}$ | $\Psi({NB}_{B}to{BR}_{R}\boldsymbol{)}$ | $\Psi({NB}_{B}to{NB}_{R}\boldsymbol{)}$ |
| $\boldsymbol{\Psi}\boldsymbol{(}\boldsymbol{NB}_{\boldsymbol{C}}\boldsymbol{to}\boldsymbol{PB}_{\boldsymbol{C}}\boldsymbol{)}$ | $\Psi({NB}_{C}to{BR}_{C}\boldsymbol{)}$ | $\Psi({NB}_{C}to{NB}_{C}\boldsymbol{)}*$ |
| $\boldsymbol{\Psi}\boldsymbol{(}\boldsymbol{NB}_{\boldsymbol{C}}\boldsymbol{to}\boldsymbol{PB}_{\boldsymbol{B}}\boldsymbol{)}$ | $\Psi({NB}_{C}to{BR}_{B}\boldsymbol{)}$ | $\Psi({NB}_{C}to{NB}_{B}\boldsymbol{)}$ |
| $\boldsymbol{\Psi}\boldsymbol{(}\boldsymbol{NB}_{\boldsymbol{C}}\boldsymbol{to}\boldsymbol{PB}_{\boldsymbol{R}}\boldsymbol{)}$ | $\Psi({NB}_{C}to{BR}_{R}\boldsymbol{)}$ | $\Psi({NB}_{C}to{NB}_{R}\boldsymbol{)}$ |

Table S2. Transition probabilities ($\Psi$) from multi-state mark recapture models that reflected general movement behaviors relative to reproductive states that were not colony-specific (
$\Psi_{StayGo}$ parameterization). Thus “goers” were birds from any of the 3 breeding colonies that transitioned between reproductive states and moved to a different colony from *year i* to *year i+1* and birds from any of the 3 colonies that transitioned between reproductive states but remained at the colony where they were resighted in the previous year were considered “stayers”.

| Constrained $\Psi$ |  |
| --- | --- |
| $\hat{\Psi}_{PR to PRstayer}*$ | $\Psi({PR}_{R}to{PR}_{R}\boldsymbol{)}$*=$\Psi({PR}_{B}to{PR}_{B}\boldsymbol{)}$*=$\Psi({PR}_{C}to{PR}_{C}\boldsymbol{)}$* |
| $\hat{\Psi}_{PR to PRgoer}$ | $\Psi({PR}_{R}to{PR}_{B}\boldsymbol{)}$=$\Psi({PR}_{R}to{PR}_{C}\boldsymbol{)}$=$\Psi({PR}_{B}to{PR}_{C}\boldsymbol{)}$=$\Psi({PR}_{B}to{PR}_{R}\boldsymbol{)}$=$\Psi({PR}_{C}to{PR}_{B}\boldsymbol{)}$=$\Psi({PR}_{C}to{PR}_{R\boldsymbol{)}}$ |
| $\hat{\Psi}_{PR to BRstayer}$ | $\Psi({PR}_{R}to{BR}_{R}\boldsymbol{)}$=$\Psi({PR}_{C}to{BR}_{C}\boldsymbol{)}$=$\Psi({PR}_{B}to{BR}_{B}\boldsymbol{)}$ |
| $\hat{\Psi}_{PR to BRgoer}$ | $\Psi({PR}_{R}to{BR}_{B}\boldsymbol{)}$=$\Psi({PR}_{R}to{BR}_{C}\boldsymbol{)}$=$\Psi({PR}_{B}to{BR}_{C}\boldsymbol{)}$=$\Psi({PR}_{B}to{BR}_{R}\boldsymbol{)}$=$\Psi({PR}_{C}to{BR}_{B}\boldsymbol{)}$=$\Psi({PR}_{C}to{BR}_{R}\boldsymbol{)}$ |
| $\hat{\Psi}_{BR to BRstayer}*$ | $\Psi({BR}_{R}to{BR}_{R}\boldsymbol{)}$*=$\Psi({BR}_{B}to{BR}_{B\boldsymbol{)}}$*=$\Psi({BR}_{C}to{BR}_{C}\boldsymbol{)}$* |
| $\hat{\Psi}_{BR to BRgoer}$ | $\Psi({BR}_{R}to{BR}_{B}\boldsymbol{)}$=$\Psi({BR}_{R}to{BR}_{C}\boldsymbol{)}$=$\Psi({BR}_{B}to{BR}_{C}\boldsymbol{)}$=$\Psi({BR}_{B}to{BR}_{R}\boldsymbol{)}$=$\Psi({BR}_{C}to{BR}_{B}\boldsymbol{)}$=$\Psi({BR}_{C}to{BR}_{R}\boldsymbol{)}$ |
| $\hat{\Psi}_{BR to NBstayer}$ | $\Psi({BR}_{R}to{NB}_{R}\boldsymbol{)}$=$\Psi({BR}_{B}to{NB}_{B}\boldsymbol{)}$=$\Psi({BR}_{C}to{NB}_{C}\boldsymbol{)}$ |
| $\hat{\Psi}_{BR to NBgoer}$ | $\Psi({BR}_{R}to{NB}_{B}\boldsymbol{)}$=$\Psi({BR}_{R}to{NB}_{C}\boldsymbol{)}$=$\Psi({BR}_{B}to{NB}_{C}\boldsymbol{)}$=$\Psi({BR}_{B}to{NB}_{R}\boldsymbol{)}$=$\Psi({BR}_{C}to{NB}_{B}\boldsymbol{)}$=$\Psi({BR}_{C}to{NB}_{R}\boldsymbol{)}$ |
| $\hat{\Psi}_{NB to BRstayer}$ | $\Psi({NB}_{R}to{BR}_{R}\boldsymbol{)}$=$\Psi({NB}_{B}to{BR}_{B}\boldsymbol{)}$=$\Psi({NB}_{C}to{BR}_{C}\boldsymbol{)}$ |
| $\hat{\Psi}_{NB to BRgoer}$ | $\Psi({NB}_{R}to{BR}_{B}\boldsymbol{)}$=$\Psi({NB}_{R}to{BR}_{C}\boldsymbol{)}$=$\Psi({NB}_{B}to{BR}_{C}\boldsymbol{)}$=$\Psi({NB}_{B}to{BR}_{R}\boldsymbol{)}$=$\Psi({NB}_{C}to{BR}_{B}\boldsymbol{)}$=$\Psi({NB}_{C}to{BR}_{R}\boldsymbol{)}$ |
| $\hat{\Psi}_{NB to NBstayer}*$ | $\Psi({NB}_{R}to{NB}_{R}\boldsymbol{)}$*=$\Psi({NB}_{B}to{NB}_{B}\boldsymbol{)}$*=$\Psi({NB}_{C}to{NB}_{C}\boldsymbol{)}$* |
| $\hat{\Psi}_{NB to NBgoer}$ | $\Psi({NB}_{R}to{NB}_{B}\boldsymbol{)}$=$\Psi({NB}_{R}to{NB}_{C}\boldsymbol{)}$=$\Psi({NB}_{B}to{NB}_{C}\boldsymbol{)}$=$\Psi({NB}_{B}to{NB}_{R}\boldsymbol{)}$=$\Psi({NB}_{C}to{NB}_{B}\boldsymbol{)}$=$\Psi({NB}_{C}to{NB}_{R}$ |

**B. Multi-state *a priori* model sets and submodeling results for** $\boldsymbol{S,p,\Psi}$

A priori model sets and model selection results for submodeling process to evaluate predicted responses for each parameter ($S,p,\Psi$) relative to general time variation ($t$), differences by sex (*SEX*), breeding colony (for $S$ and $p$ only; $R$= Royds, $B$= Bird, $C$= Crozier, and $COL$ = all colonies different), and reproductive state ($PR$=pre-breeder, $BR$=breeder, $NB$=non-breeder and $RS$= all reproductive states different) for Adelie penguins during 1999 to 2019. Transitions between reproductive states were modeled as differences between individuals that stayed at their current colony vs. those that moved to another colony during each reproductive state transition ($StayGo$). During the submodeling process we maintained a general structure on the non-modeled parameters that included the additive effects of colony (*COL*), reproductive state (*RS*), and general time variation (*t*) for $S$ and $p$. For $\Psi$, the general structure was *StayGo + t* and these structures are denoted as “$general$” in the tables below.

**Table S3**. Model selection results from the submodeling stage to evaluate the effects of colony, reproductive state, general time effects and sex on the probability of recapture ($p$).

| Model | ∆AIC*_c_^a^* | | *K* | *w_i_* | Deviance |
| --- | --- | --- | --- | --- | --- |
| $S\left( general \right)p\left( COL+RS+t+SEX \right)Psi\left( general \right)$ | 0.00 | 79 | | 0.998 | 24359.54 |
| $S\left( general \right)p\left( COL+RS+t \right)Psi\left( general \right)$ | 12.32 | 78 | | 0.002 | 24373.89 |
| $S\left( general \right)p\left( COL+RS \right)Psi\left( general \right)$ | 80.74 | 58 | | 0.000 | 24482.83 |
| $S\left( general \right)p\left( R,C=B+RS \right)Psi\left( general \right)$ | 86.39 | 57 | | 0.000 | 24490.49 |
| $S\left( general \right)p\left( RS \right)Psi\left( general \right)$ | 112.33 | 57 | | 0.000 | 24516.43 |
| $S\left( general \right)p\left( t \right)Psi\left( general \right)$ | 135.77 | 74 | | 0.000 | 24505.45 |
| $S\left( general \right)p\left( NB=PR,BR \right)Psi\left( general \right)$ | 153.52 | 55 | | 0.000 | 24561.67 |
| $S\left( general \right)p\left( R, C=B \right)Psi\left( general \right)$ | 164.08 | 56 | | 0.000 | 24570.20 |
| $S\left( general \right)p\left( COL \right)Psi\left( general \right)$ | 165.99 | 57 | | 0.000 | 24570.09 |
| $S\left( general \right)p\left( BR=NB,PR \right)Psi\left( general \right)$ | 166.73 | 55 | | 0.000 | 24574.88 |
| $S\left( general \right)p\left( R=B,C \right)Psi\left( general \right)$ | 171.49 | 56 | | 0.000 | 24577.62 |
| $S\left( general \right)p\left( SEX \right)Psi\left( general \right)$ | 178.44 | 56 | | 0.000 | 24584.57 |
| $S\left( general \right)p\left( . \right)Psi\left( general \right)$ | 180.82 | 55 | | 0.000 | 24588.96 |
| $S\left( general \right)p\left( R=C,B \right)Psi\left( general \right)$ | 182.55 | 56 | | 0.000 | 24588.67 |

^a^ Lowest AIC*_c_* = 24518.72

**Table S4**. Model selection results from the submodeling stage to evaluate the effects of colony, reproductive state, general time effects and sex on apparent survival ($S$).

| Model | ∆AIC*_c_^a^* | *K* | *w_i_* | Deviance |
| --- | --- | --- | --- | --- |
| $S\left( COL+NB=BR,PR+t \right)p\left( general \right)Psi\left( general \right)$ | 0.00 | 77 | 0.35 | 24374.35 |
| $S\left( c+NB=BR,PR+SEX+t \right)p\left( general \right)Psi\left( general \right)$ | 1.08 | 78 | 0.20 | 24373.40 |
| $SS\left( COL+RS+t \right)p\left( general \right)Psi\left( general \right)$ | 1.57 | 78 | 0.16 | 24373.89 |
| $S\left( COL+RS+SEX+t \right)p\left( general \right)Psi\left( general \right)$ | 2.63 | 79 | 0.09 | 24372.92 |
| $S\left( COL+NB=BR,PR*SEX+t \right)p\left( general \right)Psi\left( general \right)$ | 2.82 | 79 | 0.09 | 24373.11 |
| $S(R=C,B+NB=BR,PR+t)p(general)Psi(general)\}$ | 4.29 | 76 | 0.04 | 24380.67 |
| $S\left( R=C,B+NB=BR,PR+SEX+t \right)p\left( general \right)Psi\left( general \right)$ | 5.09 | 77 | 0.03 | 24379.44 |
| $S(R=C,B+RS+t)p(general)Psi(general)\}$ | 5.88 | 77 | 0.02 | 24380.23 |
| $S\left( COL+RS*SEX+t \right)p\left( general \right)Psi\left( general \right)$ | 6.40 | 81 | 0.01 | 24372.63 |
| $S\left( R=C,B+NB=BR,PB*SEX+t \right)p\left( general \right)Psi\left( general \right)$ | 6.88 | 78 | 0.01 | 24379.20 |
| $S\left( NB=BR,PB+t \right)p\left( general \right)Psi\left( general \right)$ | 20.68 | 75 | 0.00 | 24399.08 |
| $S\left( RS+t \right)p\left( general \right)Psi\left( general \right)$ | 22.13 | 76 | 0.00 | 24398.51 |
| $S\left( t \right)p\left( general \right)Psi\left( general \right)$ | 211.74 | 74 | 0.00 | 24592.17 |
| $S\left( NB=BR,PB \right)p\left( general \right)Psi\left( general \right)$ | 296.38 | 56 | 0.00 | 24713.25 |
| $S\left( RS \right)p\left( general \right)Psi\left( general \right)$ | 297.88 | 57 | 0.00 | 24712.73 |
| $S\left( NB=PR,BR \right)p\left( general \right)Psi\left( general \right)$ | 404.79 | 56 | 0.00 | 24821.66 |
| $S\left( R=C,B \right)p\left( general \right)Psi\left( general \right)$ | 508.24 | 56 | 0.00 | 24925.12 |
| $S\left( COL \right)p\left( general \right)Psi\left( general \right)$ | 509.51 | 57 | 0.00 | 24924.37 |
| $S\left( SEX \right)p\left( general \right)Psi\left( general \right)$ | 538.51 | 56 | 0.00 | 24955.38 |
| $S\left( R=B,C \right)p\left( general \right)Psi\left( general \right)$ | 540.26 | 56 | 0.00 | 24957.14 |
| $S\left( . \right)p\left( general \right)Psi\left( general \right)$ | 540.69 | 55 | 0.00 | 24959.58 |
| $S\left( R, B=C \right)p\left( general \right)Psi\left( general \right)$ | 540.99 | 56 | 0.00 | 24957.87 |

^a^ Lowest AIC*_c_* = 24529.47

**Table S5**: Model selection results from the submodeling stage to evaluate the effects of time and sex on the probability of transitioning between states ($\Psi$) for birds that also move between colonies and those that do not (*StayGo*).

| Model | ∆AIC*_c_^a^* | *K* | *w_i_* | Deviance |
| --- | --- | --- | --- | --- |
| $S\left( general \right)p\left( general \right)Psi\left( StayGo*SEX+t \right)$ | 0.00 | 87 | 0.88 | 24330.72 |
| $S\left( general \right)p\left( general \right)Psi\left( StayGo+SEX+t \right)$ | 3.99 | 79 | 0.12 | 24350.96 |
| $S\left( general \right)p\left( general \right)Psi\left( StayGo+t \right)$ | 24.90 | 78 | 0.00 | 24373.89 |
| $S\left( general \right)p\left( general \right)Psi\left( StayGo*SEX \right)$ | 167.58 | 67 | 0.00 | 24538.88 |
| $S\left( general \right)p\left( general \right)Psi\left( StayGo+SEX \right)$ | 171.91 | 59 | 0.00 | 24559.39 |
| $S\left( general \right)p\left( general \right)Psi\left( StayGo \right)$ | 493.54 | 58 | 0.00 | 24883.05 |

^a^ Lowest AIC*_c_* = 24506.14

**C. Hypothetical sex ratios for a single cohort of 2-year-old Adelie penguins, given sex-specific recruitment rates and survival relative to breeding state (pre-breeders vs. breeders) estimated for known-sex Ross Island penguins, 1998-2020.**

**Table S6**: Number of pre-breeders, and new recruits (breeders) by age class starting with 1000 male (a) and 1000 female (b) 2-year-olds at *time i*, with annual survival of pre-breeders = 0.90 (S_PB_) for both sexes, and sex-specific probabilities of recruiting into the breeding population at each age class (Y_PBtoBR_= 0.247 Males; 0.323 for females).

Numbers calculated as follows:

Column A: Number of pre-breeders, age @ *time i*: age 2 = 1000 & subsequent age @ *time i* = (Column B – Column C)

Column B: Number of pre-breeders, age @ *time i+1* = Column A * (S_PB)_;

Column C: Number of breeders, age @ *time i+1* = Column B * (Ψ_PBtoBR_)

a). Males

| Age_@ time i_ | Age_@ time i+1_ | **A**  N_PB age @ time i_ | S_PB_ | **B**  N_PB age @ time i+1_ | Ψ_PBtoBR_ | **C**  N_BR age @ time i+1_ |
| --- | --- | --- | --- | --- | --- | --- |
| 2 | 3 | 1000 | 0.90 | 902 | 0.247 | 223 |
| 3 | 4 | 679 | 0.90 | 612 | 0.247 | 151 |
| 4 | 5 | 461 | 0.90 | 416 | 0.247 | 103 |
| 5 | 6 | 313 | 0.90 | 282 | 0.247 | 70 |
| 6 | 7 | 213 | 0.90 | 192 | 0.247 | 47 |
| 7 | 8 | 144 | 0.90 | 130 | 0.247 | 32 |
| 8 | 9 | 98 | 0.90 | 88 | 0.247 | 22 |
| 9 | 10 | 67 | 0.90 | 60 | 0.247 | 15 |

b). Females

| Age_@ time i_ | Age_@ time i+1_ | **A**  N_PB age @ time i_ | S_PB_ | **B**  N_PB age @ time i+1_ | Ψ_PBtoBR_ | **C**  N_BR age @ time i+1_ |
| --- | --- | --- | --- | --- | --- | --- |
| 2 | 3 | 1000 | 0.90 | 902 | 0.323 | 291 |
| 3 | 4 | 611 | 0.90 | 551 | 0.323 | 178 |
| 4 | 5 | 373 | 0.90 | 336 | 0.323 | 109 |
| 5 | 6 | 228 | 0.90 | 205 | 0.323 | 66 |
| 6 | 7 | 139 | 0.90 | 125 | 0.323 | 41 |
| 7 | 8 | 85 | 0.90 | 77 | 0.323 | 25 |
| 8 | 9 | 52 | 0.90 | 47 | 0.323 | 15 |
| 9 | 10 | 32 | 0.90 | 29 | 0.323 | 9 |

**Table S7**: Total number of males (a) and females (b) and resulting ASR (c) for a single hypothetical cohort of 2000, 2-year-olds with an even sex ratio (i.e., ASR = 0.50 at age 2). Numbers of pre-breeders and new recruits at age *time i+1* and surviving breeders for age at *time i+1* from previous recruitment year (i.e., *time i*).

a). Males

| Age @ *time i+1* | N_BR_ | N_PB_ | N_BR survived from age i_ | Total Males |
| --- | --- | --- | --- | --- |
| 3 | 223 | 679 | 0 | 902 |
| 4 | 151 | 461 | 178 | 791 |
| 5 | 103 | 313 | 121 | 537 |
| 6 | 70 | 213 | 82 | 365 |
| 7 | 47 | 144 | 56 | 248 |
| 8 | 32 | 98 | 38 | 168 |
| 9 | 22 | 67 | 26 | 114 |
| 10 | 15 | 45 | 17 | 77 |

b). Females

| Age @ *time i+1* | N_BR_ | N_PB_ | N_BR survived from age i_ | Total Females |
| --- | --- | --- | --- | --- |
| 3 | 291 | 611 | 0 | 902 |
| 4 | 178 | 373 | 233 | 784 |
| 5 | 109 | 228 | 142 | 478 |
| 6 | 66 | 139 | 87 | 292 |
| 7 | 40 | 85 | 53 | 178 |
| 8 | 25 | 52 | 32 | 109 |
| 9 | 15 | 32 | 20 | 66 |
| 10 | 9 | 19 | 12 | 41 |

c). Adult sex ratio as the proportion of males in the population (i.e., total males/(total males + total females))

| Age @ *time i+1* | ASR |
| --- | --- |
| 3 | 0.50 |
| 4 | 0.50 |
| 5 | 0.53 |
| 6 | 0.56 |
| 7 | 0.58 |
| 8 | 0.61 |
| 9 | 0.63 |
| 10 | 0.66 |
